# National incidence of autoimmune liver diseases and its relationship with the human development index

**DOI:** 10.18632/oncotarget.10090

**Published:** 2016-06-15

**Authors:** Hong-Ying Pan, Yi-Ning Dai, Ji-Na Zheng, Ke-Qing Shi, Sven Van Poucke, Hai Zou, Ming-Hua Zheng

**Affiliations:** ^1^ Department of Infection Diseases, Zhejiang Provincial People's Hospital, Hangzhou, China; ^2^ Department of Hepatology, Liver Research Center, The First Affiliated Hospital of Wenzhou Medical University, Wenzhou, China; ^3^ School of The First Clinical Medical Sciences, Wenzhou Medical University, Wenzhou, China; ^4^ Institute of Hepatology, Wenzhou Medical University, Wenzhou, China; ^5^ Department of Anesthesiology, Intensive Care, Emergency Medicine and Pain Therapy, Ziekenhuis Oost-Limburg, Genk, Belgium

**Keywords:** autoimmune liver diseases, human development index, autoimmune hepatitis, primary biliary cirrhosis, primary sclerosing cholangitis

## Abstract

**Objective:**

Primary biliary cirrhosis (PBC), primary sclerosing cholangitis (PSC), autoimmune hepatitis (AIH) and immunoglobulin G4 related cholangitis represent the major autoimmune liver diseases (AILD). However, the relationship between AILD incidence and socioeconomic development levels is yet to be explored.

**Results:**

A total of 43 studies were included. There was a positive but not significant correlation between the PBC incidence and HDI on a global level (r=0.348, *P*=0.082). However, in Europe, a significantly positive correlation existed between the PBC incidence and HDI (r=0.455, *P*=0.044). No statistical correlation between PSC incidence and HDI was observed (r=0.116, *P*=0.706). The incidence of AIH revealed a positive correlation with the national HDI both globally (r=0.638, *P*=0.014) and in Europe (r=0.644, *P*=0.045). Moreover, the PBC incidence demonstrated a positive correlation with the health index (r=0.422, *P*=0.036), but a negative correlation with the education index (r= −0.650, *P*<0.01). Moreover, the income index presented a positive correlation with both the PSC incidence (r=0.599, *P*=0.031) and the AIH incidence (r=0.649, *P*=0.012).

**Methods:**

PubMed was searched to identify relevant epidemiological studies on AILD. The human development index (HDI) was applied as an indicator for socioeconomic development. HDI data were obtained and calculated based on the 2014 Human Development Report. Pearson coefficient and linear regression analysis were conducted to estimate the correlation between incidence and HDI.

**Conclusions:**

There is positive association between the national incidence of AILD and the socioeconomic status, as measured by HDI. In less-developed countries, the incidence of AILD, especially PBC and AIH, might be less common.

## INTRODUCTION

Autoimmune liver diseases (AILD) cover a spectrum of primary biliary cirrhosis (PBC), primary sclerosing cholangitis (PSC), autoimmune hepatitis (AIH) and immunoglobulin G4 (IgG4) related cholangitis. These are all relatively rare diseases but result in significant morbidity and mortality. A considerable proportion of patients with AILD eventually progress to end-stage liver disease. In Europe, AILD represent a major indication for liver transplantation. As complex disorders, AILD develop from the interactions between a variety of genetic and environmental factors [1–3]. Until now, the pathogenesis of PBC, PSC, AIH or IgG4 related cholangitis has not been fully elaborated.

Previous epidemiological studies have reported that the incidence of AILD varies over time and space [4]. It is widely accepted that, in genetically susceptible individuals, environmental risk factors might contribute to the onset of disease [2]. The rapid economical development of society leads to changes in lifestyle, hygienic and psychosocial conditions. The influence of the socioeconomic status on the incidence of AILD has never been studied. Herein, we systematically reviewed epidemiological studies of AILD from all over the world, and explored the relationship between the AILD incidence and the socioeconomic status. In this study, the human development index (HDI) was applied as an indicator to reflect socioeconomic status. We are convinced that this study might provide additional insight into the pathogenesis and etiology of these diseases.

## RESULTS

### Study characteristics

Among the 43 enrolled studies, 22 reported the incidence of PBC [5–26], while 13 were relevant for PSC [13-14, 17, 27-36], and 14 provided information about epidemiology of AIH [4, 13-14, 17, 37-46]. No studies mentioned the population incidence of IgG4 related cholangitis, presumably because it is relatively rare as a new disease entity.

Primary characteristics of the enrolled studies including first author, publication year, research period, country, continent, criteria of disease identification, incidence (per 100,000 per year) and HDI were summarized in Table 1. A majority of the studies (29 of 42) were conducted in Europe, while six studies were conducted in North America, six and two were conducted in Asia and Australia, respectively.

**Table 1:** Primary characteristics of the included studies

Study	Publication year	Disease	Disease identification	Country	Continent	Research period	Incidence per 100,000 per year	HDI
Triger^8^	1980	PBC	Cholestasis; AMA	United Kingdom	Europe	1977-1979	0.58	0.735
Eriksson^9^	1984	PBC	Cholestasis; AMA	Sweden	Europe	1973-1982	1.37	0.776
Borda^10^	1989	PBC	Cholestasis; AMA	Spain	Europe	1974-1987	1974: 0.41 1987: 2.52	1974: NA 1987: 0.755
Myszor^11^	1990	PBC	Cholestasis; AMA; liver biopsy	United Kingdom	Europe	1965-1987	1987: 1.98	1987: 0.768
Danielsson^12^	1990	PBC	(Cholestasis + AMA) or liver biopsy	Sweden	Europe	1973-1982	1.33	0.776
Almdal^13^	1991	PBC	Registry-based	Denmark	Europe	1981-1985	0.90	0.788
Remmel^14^	1995	PBC	(Cholestasis + AMA) or liver biopsy	Estonia	Europe	1973-1992	0.23	0.684
Metcalf^15^	1997	PBC	2 of 3: AMA positive; abnormal liver function; liver biopsy	United Kingdom	Europe	1987-1994	2.20	0.839
Berdal^16^	1998	PBC	Cholestasis; AMA; liver biopsy	Norway	Europe	1985-1994	1.20	0.843
Berdal^16^	1998	PSC	Cholangiography; exclusion of alternatives	Norway	Europe	1985-1994	0.70	0.843
Berdal^16^	1998	AIH	Original IAHG criteria^6^	Norway	Europe	1985-1994	1.60	0.843
Boberg^17^	1998	PBC	Cholestasis; AMA; liver biopsy	Norway	Europe	1986-1995	1.62	0.849
Boberg^17^	1998	PSC	Cholestasis; Cholangiography; liver biopsy	Norway	Europe	1986-1995	1.31	0.849
Boberg^17^	1998	AIH	Original IAHG criteria^6^	Norway	Europe	1986-1995	1.90	0.849
James^18^	1999	PBC	2 of 3: AMA positive; abnormal liver function; liver biopsy	United Kingdom	Europe	1987-1994	1987: 2.30 1994: 3.22	1987: 0.758 1994: 0.806
Kim^19^	2000	PBC	(Cholestasis + AMA) or liver biopsy	USA	North America	1975-1995	2.70	0.847
Whalley^20^	2007	PBC	Cholestasis; AMA	United Kingdom	Europe	2003-2004	3.50	0.881
Whalley^20^	2007	PSC	Cholangiography	United Kingdom	Europe	2003-2004	2.00	0.881
Whalley^20^	2007	AIH	Abnormal liver function; presence of autoantibodies	United Kingdom	Europe	2003-2004	3.00	0.881
Rautiainen^21^	2007	PBC	2 of 3: Cholestasis; AMA positive; liver biopsy	Finland	Europe	1988-1999	1988: 1.20 1999: 1.70	1988: 0.784 1999: 0.836
Pla^22^	2007	PBC	2 of 3: Cholestasis; AMA positive; liver biopsy	Spain	Europe	1990-2002	1.72	0.794
Myers^23^	2009	PBC	Cholestasis; AMA	Canada	North America	1996-2002	3.03	0.868
Chong^24^	2010	PBC	2 of 3: Cholestasis; AMA positive; liver biopsy	Brunei	Asia	2007	1.03	0.841
Delgado^25^	2012	PBC	2 of 3: Cholestasis; AMA positive; liver biopsy	Israel	Asia	1990-2010	1990-1999: 1.00 2000-2010: 2.00	1990-1999: 0.814 2000-2010: 0.865
Baldursdottir^26^	2012	PBC	2 of 3: Cholestasis; AMA positive; liver biopsy	Iceland	Europe	1991-2010	1991-2000: 2.00 2001-2010: 2.50	1991-2000: 0.829 2001-2010: 0.872
Ngu^27^	2012	PBC	2 of 3: Cholestasis; AMA positive; liver biopsy	New Zealand	Australia	2008	0.80	0.899
Koulentaki^28^	2014	PBC	2 of 3: Cholestasis; AMA positive; liver biopsy	Greece	Europe	1990-2010	2.09	0.803
Boonstra^29^	2014	PBC	2 of 3: Cholestasis; AMA positive; liver biopsy	Netherlands	Europe	2000-2007	1.10	0.885
Escorsell^30^	1994	PSC	Cholangiography	Spain	Europe	1984-1988	0.04	0.733
Hurlburt^31^	2002	PSC	Cholangiography	USA	North America	1984-2000	0	0.861
Ang^32^	2002	PSC	Cholangiography; exclusion of alternatives	Singapore	Asia	1989-1998	1.33	0.767
Bambha^33^	2003	PSC	Cholestasis; Cholangiography or liver biopsy; exclusion of alternatives	USA	North America	1976-2000	0.90	0.854
Kingham^34^	2004	PSC	Abnormal liver function; Cholangiography; liver biopsy	United Kingdom	Europe	1984-2003	0.91	0.813
Kaplan^35^	2007	PSC	Abnormal liver function; Cholangiography or liver biopsy; exclusion of alternatives	Canada	North America	2000-2005	0.92	0.880
Card^36^	2008	PSC	Registry-based	United Kingdom	Europe	1990-2001	0.41	0.876
Lindkvist^37^	2010	PSC	Cholestasis; Cholangiography or liver biopsy; exclusion of alternatives	Sweden	Europe	1992-2005	1.22	0.855
Toy^38^	2011	PSC	Cholangiography or liver biopsy	USA	North America	2000-2006	0.41	0.897
Boonstra^39^	2013	PSC	Cholestasis; Cholangiography or liver biopsy; exclusion of alternatives	Netherlands	Europe	2000-2007	0.50	0.885
Ritland^40^	1985	AIH	Abnormal liver function; presence of autoantibodies; exclusion of alternatives	Norway	Europe	1981	1.19	0.798
Tanner^41^	1989	AIH	Abnormal liver function; liver biopsy; exclusion of alternatives	United Kingdom	Europe	1971-1987	0.42	0.735
Lee^42^	2001	AIH	Original IAHG criteria^6^	Singapore	Asia	1990-1996	0.60	0.761
Primo^43^	2004	AIH	Original^6^, revised^7^, or simplified criteria^5^	Spain	Europe	1990-2003	0.83	0.796
Werner^44^	2008	AIH	Revised IAHG criteria^7^	Sweden	Europe	1990-2003	0.85	0.848
Primo^45^	2009	AIH	Revised IAHG criteria^7^	Spain	Europe	2003	1.07	0.837
Ngu^46^	2010	AIH	Revised^7^ or simplified criteria^5^	New Zealand	Austrilia	2008	1.70	0.899
Delgado^4^	2013	AIH	Simplified criteria^5^	Israel	Asia	1995-2010	0.67	0.849
Grønbæk^47^	2014	AIH	Registry-based	Denmark	Europe	1994-2012	1.68	0.864
Van Gerven^48^	2014	AIH	Revised^7^ or simplified criteria^5^	Netherlands	Europe	2000-2010	1.10	0.889
Yoshizawa^49^	2015	AIH	Revised IAHG criteria	Japan	Asia	2004-2014	1.52	0.882

HDI: human development index; PBC: primary biliary cirrhosis; PSC: primary sclerosing cholangitis; AIH: autoimmune hepatitis; AMA: anti-mitochondrial antibodies; IAHG: international autoimmune hepatitis group; NA: not available.

The national incidence of AILD varied among the studies performed in different countries or during different study periods. A relatively higher incidence of disease was associated with a higher national HDI. However, the included studies were generally conducted in countries with higher socioeconomic development. A total of 28 studies were performed in countries with very high HDI, 14 in high HDI countries, and one in medium HDI country. Few studies investigated the incidence of AILD in less developed countries with medium or low HDI.

### PBC incidence and HDI

**Figure 1:**
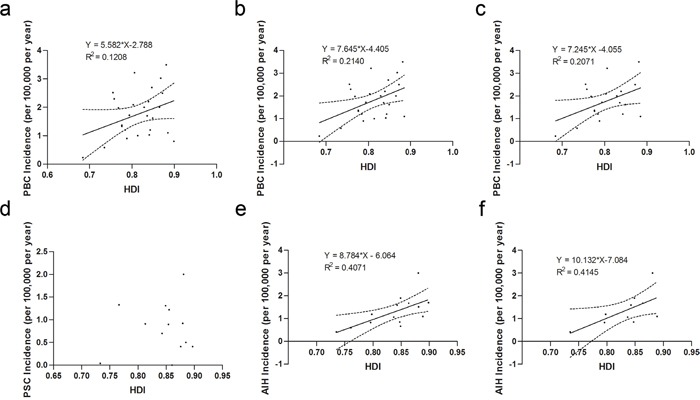
National incidence of autoimmune liver diseases and human development index **a.** Overall incidence of PBC (r = 0.348, *P* = 0.082); **b.** Incidence of PBC after exclusion of the study conducted in New Zealand (r = 0.463, *P* = 0.020); **c.** Incidence of PBC in Europe (r = 0.455, *P* = 0.044); **d.** Incidence of PSC (r = 0.116, *P* = 0.706); **e.** Overall incidence of AIH (r = 0.638, *P*= 0.019); **f.** Incidence of AIH in Europe (r = 0.644, *P* = 0.045). Best-fit lines (solid) and the 95% confidence band of the best-fit line (dashed) by linear regression are indicated.

The incidence of PBC was positively correlated with the national HDI according to Pearson correlation and linear regression analysis, but there was no statistical significance [β = 5.582 ± 6.343 (95% CI), r = 0.348, *P* = 0.082] (Figure 1a, Table 2). We noticed that the incidence of PBC in New Zealand, Australia was particularly low compared to those reported in other locations for a similar period.[24] After removing the study by Ngu et al.,[24] a significantly positive correlation between HDI and PBC incidence was observed [β = 7.645 ± 6.320 (95% CI), r = 0.463, *P* = 0.020] (Figure 1b).

**Table 2:** Correlation between AILD incidence and HDI, along with its three dimensions

Disease	Parameters	HDI	Health Index	Education index	Income Index
PBC	R	0.348	0.422	−0.650	−0.284
	β	5.582	2.193	−2.370	−0.131
	95% CI of β	[−0.761, 1.925]	[0.159, 4.227]	[−3.565, −1.175]	[−0.316, 0.055]
	P	0.082	0.036	< 0.01	0.159
PSC	R	0.116	0.180	0.235	0.599
	β	1.355	2.605	0.761	5.285
	95% CI of β	[−6.346, 9.055]	[−6.838, 12.049]	[−1.325, 2.847]	[0.594, 9.976]
	P	0.706	0.556	0.439	0.031
AIH	R	0.638	0.187	0.219	0.649
	β	8.784	2.165	0.916	7.575
	95% CI of β	[2.116, 15.452]	[−4.972, 9.302]	[−1.656, 3.488]	[1.987, 13.164]
	P	0.014	0.521	0.453	0.012

AILD: autoimmune liver disease; HDI: human development index; PBC: primary biliary cirrhosis; PSC: primary sclerosing cholangitis; AIH: autoimmune hepatitis; CI: confidence interval.

In subgroup analysis for geographic location (continent), Figure 1c demonstrated that the PBC incidence in Europe was statistically, positively correlated with the national HDI [β = 7.245 ± 7.021 (95% CI), r = 0.455, *P* = 0.044]. The relationship between the HDI and PBC incidence in Asia, North America or Australia was impossible to be identified due to the limited number of studies involved.

### PSC incidence and HDI

There was no correlation between the PSC incidence and the national HDI (r = 0.116, *P* = 0.706) (Figure 1d, Table 2). Subsequent subgroup analysis for geographic location (continent) indicated no correlation between the incidence of PSC and HDI in Europe (r = 0.508, *P* = 0.198) or in North America (r = −0.030, *P* = 0.970).

### AIH incidence and HDI

We found a significantly positive correlation between the national HDI and AIH incidence [β = 8.784 ± 6.668 (95% CI), r = 0.638, *P* = 0.014] (Figure 1e, Table 2). In Europe, the national incidence of AIH was positively correlated with the HDI [β = 10.132 ± 9.817 (95% CI), r = 0.644, *P* = 0.045] (Figure 1f). Limited data prevented further analysis about this relationship in other continents.

### Disease incidence in countries with different HDIs

**Figure 2:**
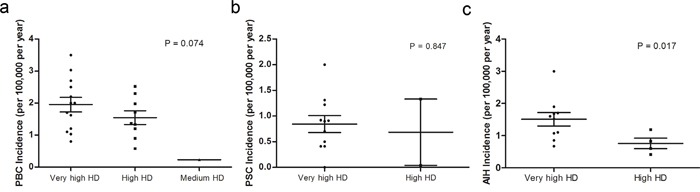
National incidence of autoimmune liver diseases in countries with different development levels **a.** Incidence of PBC; **b.** Incidence of PSC; **c.** Incidence of AIH. One-way analysis of variance or unpaired t test was conducted in the comparison.

Studies investigating the PBC incidence were classified into three groups based on the fact if they were conducted in very high, high or medium HDI countries. ANOVA analysis revealed no significant difference among the groups (*P* = 0.074) (Figure 2a). Countries with a very high HDIs presented a mean PBC incidence of 1.981 per 100,000 per year (Standard deviation (SD): 0.842), while high HDI countries had a mean PBC incidence of 1.544 per 100,000 per year (SD: 0.641). Similarly, we removed the study conducted in New Zealand, Australia by Ngu et al.,[24] and found that PBC was significantly more prevalent in countries with higher HDIs (*P* = 0.045 by ANOVA).

Compared to countries with very high HDIs (mean incidence: 0.844 per 100,000 per year, SD: 0.542), high HDI countries had no difference of PSC incidence (mean incidence: 0.685 per 100,000 per year, SD: 0.912) (*P* = 0.847 by unpaired t test) (Figure 2b).

Very high HDI countries had a mean AIH incidence of 1.509 per 100,000 per year (SD: 0.662), significantly higher than in countries with high HDIs (mean incidence: 0.760 per 100,000 per year, SD: 0.332) (*P* = 0.017 by unpaired t test) (Figure 2c).

### Disease incidence with three dimensions of HDI

We further evaluated the correlations between the incidence of AILD and the three dimensions of HDI (Table 2). Interestingly, the incidence of PBC was positively correlated with life expectancy at birth (health index) [β = 2.193 ± 2.034 (95% CI), r = 0.422, *P* = 0.036], but showed a negative correlation with the education index [β = −2.370 ± 1.195 (95% CI), r = −0.650, *P* < 0.01]. There was a positive correlation between the PSC incidence and the income index [β = 5.285 ± 4.691 (95% CI), r = 0.599, *P* = 0.031]. In terms of AIH, the incidence also presented a positive correlation with the income index [β = 7.575 ± 5.589 (95% CI), r = 0.649, *P* = 0.012].

## DISCUSSION

This study revealed that socioeconomic development, as indicated by the HDI, might have a positive correlation with the incidence of PBC and AIH. Countries with very high HDI tended to have a higher incidence of PBC and AIH compared to countries with high or medium HDI. In addition, the economic status, as indicated by GNI per capita (income index of HDI), had a positive correlation with the incidence of PSC and AIH. We additionally found some association between national incidence of AILD and the socioeconomic status.

It has been commonly recognized that AILD, mainly consisted of PBC, PSC and AIH, develops as a result from the influence of multiple genes in combination with currently undefined environmental factors. Environmental risk factors might be very complex, but are regarded to trigger the disease onset. PBC is a female predominant liver disease. A controlled interview-based study in the United States revealed that a history of urinary tract infections, cigarette smoking, use of hormone replacement therapies, or frequent use of nail polish were associated with an increased risk for PBC [47]. On the other hand, PSC is a progressive cholestatic liver disease, which is strongly associated with inflammatory bowel disease. A case-control study in the Netherlands has demonstrated that smoking has a protective effect for developing PSC [1]. In terms of AIH, it is mentioned that alcohol drinking and living in a childhood home with wood heating are protective factors against the development of AIH [2]. Other putative environmental factors of AILD include keeping pets, type of housing, number of siblings and family manners. An unidentified agent (i.e., toxin/microbe) might be involved in the development of AILD [3]. Tenca et al. have confirmed that close contact with a pet (especially a cat) during childhood increase the risk of AILD [3]. All these environmental factors as mentioned above might be related to the development of socioeconomic status and subsequent lifestyle changes. Based on this correlation, we made a hypothesis that the incidence of AILD might have a causal relationship with human socioeconomic development.

In this study, a comprehensive review is presented, covering all available relevant studies that report the incidence of AILD without restriction of geographic region or language. On a global level, there was a positive but not significant correlation between PBC incidence and HDI. When excluding the study by Ngu et al conducted in New Zealand [24], whose PBC incidence was especially low compared to other locations in a similar research period, the incidence of PBC demonstrated a positive correlation with HDI. In addition, the PBC incidence in Europe correlated positively with HDI. We found a similar positive correlation in regard to the AIH incidence and the HDI, both worldwide and in Europe. However, we failed to get the similar result in terms of PSC.

A deep analysis on the three dimensions of HDI has been performed. Interestingly, a high GNI per capita had a close association with PSC and AIH prevalence. Furthermore, a higher PBC incidence was related with a higher life expectancy at birth and a lower mean and expected years of schooling. These results summarized in our study might offer clues to the pathogenesis of AILD.

It is the first time a research was conducted, investigating the relationship between the AILD incidence and socioeconomic development. The HDI was selected as the measurement tool because of its worldwide acceptance in international comparisons of development levels. Another strength of this study is that we set up rigorous inclusion criteria, including only population-based studies with definite diagnosis of AILD. Population-based surveys have higher validity because everyone in the at-risk population, or a random sample of the population, is accounted for.

Nevertheless, a few limitations of this study must be taken into consideration. First of all, the review of AILD epidemiology was confined to Europe, North America, Asia and Australia, due to limited data in Africa and Oceania. Secondly, different areas within a country usually have different development levels. However, HDI is the summary of the average socioeconomic status of a country. Moreover, some studies were conducted in a specific region or a single province of a country, which resulted in an uneven distribution of incidence data. Thirdly, we failed to make subgroup analyses according to gender, ethnicity or region (rural/urban gap), on account of the insufficient data. In some way, different populations in an area might have variations in the disease incident rates.

In summary, we have demonstrated a potentially positive relationship between the national incidence of AILD and HDI. This study provides for the first time the evidence that in areas with higher development levels, AILD might be more prevalent, and offers a distinctive perspective on the global epidemiological status of AILD. A more robust and comprehensive analysis is required for further more epidemiological investigations in other continents and based on more detailed information.

## MATERIALS AND METHODS

### Diagnosis of autoimmune liver diseases

For the purpose of this paper, only studies were included with definite criteria for the diagnosis of autoimmune liver disease. The diagnostic criteria for PBC included: (i) clinical presentation; (ii) elevated serum level of alkaline phosphatase of liver origin for more than 6 months; (iii) presence of serum anti-mitochondrial antibodies (≥1:40); (iv) histological characteristics of florid bile duct lesions. Criteria (ii) and (iii) were diagnostic for PBC, and criteria (iv) confirmed the diagnosis.

The identification of PSC was based on increased serum levels of alkaline phosphatase and γ-glutamyltranspeptidase not otherwise explained, when magnetic resonance cholangiography (MRC) or endoscopic retrograde cholangiography (ERC) demonstrated bile duct changes with multifocal strictures and segmental dilatations. Secondary causes of sclerosing cholangitis and other cholestatic disorders were excluded.

AIH was diagnosed based on the combination of clinical, biochemical (e.g. presence of autoantibodies, elevated immunoglobulin G level) and histological conditions according to the reported criteria [48–50].

### Definition and calculation of HDI

The HDI is a compound measure of average achievement in three key dimensions of human development: a long and healthy life (health index: life expectancy at birth), being knowledgeable (education index: mean and expected years of schooling) and having a decent standard of living (income index: gross national income (GNI) per capita).

The HDI data were obtained from the United Nations Development Programme database (http://hdr.undp.org/en/data). The average HDI over the research period was calculated, and was defined as the HDI for each study. We hypothesized that HDI progressed linearly in a short period of time and estimated the missing HDI data based on the available data. According to the 2014 Human Development Report (HDR 2014), quartiles of HDI distribution were defined as follows: very high human development (HDI ≥ 0.800), high human development (0.700 ≤ HDI< 0.800), medium human development (0.550 ≤HDI< 0.700), and low human development (HDI < 0.550).

### Search strategy and literature evaluation

Literature was searched using PubMed from inception to January 2016 to identify relevant national epidemiological studies of autoimmune liver disease. No language restriction was imposed. Search terms were as follows: (“Liver Cirrhosis, Biliary/epidemiology”[Mesh] OR “primary biliary cirrhosis” OR “primary biliary cholangitis” OR “Cholangitis, Sclerosing/epidemiology”[Mesh] OR “primary sclerosing cholangitis” OR “primary sclerotic cholangitis” OR “Hepatitis, Autoimmune/epidemiology”[Mesh] OR “autoimmune hepatitis” OR “G4 related cholangitis” OR “G4 associated cholangitis”) AND (prevalence OR incidence OR epidemic OR morbidity).

Only studies that present incidence of PBC, PSC or AIH with a definite diagnosis in an adult population were included. Non population-based surveys were disqualified, such as those only enrolled patients in a particular hospital or participants taking part in a particular health plan.

Two investigators independently completed eligibility evaluation and data extraction. Disagreement was solved by discussion with the third investigator. A total of 1708 records were identified through basic search. 1652 publications were excluded after screening through titles and abstracts. Further eligibility evaluation resulted in the exclusion of nine studies because of the absence of data about HDI or disease incidence, and an additional exclusion of four studies because they were not adult population-based. Finally, a total of 43 surveys were included in this study.

### Statistical analysis

All statistical analysis was performed by SPSS 21.0 (IBM, Chicago, IL, USA). Associated data was plotted using GraphPad Prism (6.0, GraphPad Software, Inc., San Diego, CA, USA). Kolmogorov–Smirnoff test was conducted to examine the normal distribution of variables. Correlations between the incidence of AILD and HDI were obtained by Pearson coefficient (with normal distribution); in cases of skewed distributions of variables, the Spearman coefficient was applied. In addition, a linear regression analysis was conducted. One-way analysis of variance (ANOVA) or an unpaired t-test was used to compare disease incidence in groups with different socioeconomic development. A *P* value of less than 0.05 was considered as statistically significant.

## Abbreviations

PBC: primary biliary cirrhosis
PSC: primary sclerosing cholangitis
AIH: autoimmune hepatitis
AILD: autoimmune liver diseases
HDI: human development index
IgG4: immunoglobulin G4
MRC: magnetic resonance cholangiography
ERC: endoscopic retrograde cholangiography
GNI: gross national income
HDR: human development report
IBD: inflammatory bowel disease.
